# A New Approach of *In Vivo* Musculoskeletal Tissue Engineering Using the Epigastric Artery as Central Core Vessel of a 3-Dimensional Construct

**DOI:** 10.1155/2012/510852

**Published:** 2012-02-28

**Authors:** Sebastian E. Dunda, T. Schriever, C. Rosen, C. Opländer, R. H. Tolba, S. Diamantouros, S. Jockenhoevel, N. Pallua

**Affiliations:** ^1^Department of Plastic Surgery, Reconstructive and Hand Surgery, Burn Center, University Hospital Aachen, RWTH, Pauwelsstraße 30, 52074 Aachen, Germany; ^2^Department of Anatomy, University Hospital Aachen, 52074 Aachen, Germany; ^3^Institute of Laboratory Animal Science and Experimental Surgery, University Hospital Aachen, 52074 Aachen, Germany; ^4^Helmholtz Institute for Biomedical Engineering, RWTH Aachen, 52074 Aachen, Germany

## Abstract

The creation of musculoskeletal tissue represents an alternative for the replacement of soft tissue in reconstructive surgery. However, most of the approaches of creating artificial tissue have their limitations in the size as the maximally obtainable dimension of bioartificial tissue (BAT) is limited due to the lack of supporting vessels within the 3-dimensional construct. The seeded myoblasts require high amounts of perfusion, oxygen, and nutrients to survive. To achieve this, we developed a 3-dimensional scaffold which features the epigastric artery as macroscopic core vessel inside the BAT in a rat model (perfused group, *n* = 4) and a control group (*n* = 3) without the epigastric vessels and, therefore, without perfusion. The *in vivo* monitoring of the transplanted myoblasts was assessed by bioluminescence imaging and showed both the viability of the epigastric artery within the 3-dimensional construct and again that cell survival *in vivo* is highly depending on the blood supply with the beginning of capillarization within the BAT seven days after transplantation in the perfused group. However, further studies focussing on the matrix improvement will be necessary to create a transplantable BAT with the epigastric artery as anastomosable vessel.

## 1. Introduction

The replacement and reconstruction of musculoskeletal tissue after severe damage caused by traumatic injury, tumor surgery, or prolonged denervation is limited with the utilizable number of transplantable muscle tissue. Moreover, the transfer of muscle tissue is not uncommonly associated with aesthetic and functional impairment at the donor site. Thus, musculoskeletal tissue engineering is attempting to be an alternative in the challenging field of reconstructive surgery [1–5]. However, during the last decade of stem cell and tissue engineering research, it turned out that one main critical factor in tissue engineering is the lack of supporting blood vessels and insufficient capillarization [6, 7]. Therefore different *in vivo* models like the AV-loop using the femoral vessels have been developed and confirmed a much higher cell survival by inducing vascularisation of the used matrix and furthermore the possibility to create a 3D-bioartificial tissue construct [8–14]. In this study, we established a new *in vivo* model of musculoskeletal tissue engineering using the inferior and superior epigastric artery as central-core vessels of our custom-made implantable bioreactor chamber. In order to follow-up our transplanted myoblasts *in vivo*, the cells have been transfected with Luciferase and could in this way be atraumatically monitored with bioluminescence [15–17]. 

## 2. Materials and Methods

### 2.1. Myoblasts Cell Culture

Myoblasts were harvested by dissecting the soleus and gastrocnemius muscles of 5 days old male Wistar rats (Charles River, Kisslegg, Germany) as described by Bach et al. [12]. Satellite cells were obtained from the minced muscles by digestion with 0.1% collagenase type III (Biochrom, Berlin, Germany) for 60 minutes and 0.25% trypsin (Viralex, PAA Laboratories, Linz, Austria) for 45 minutes at 37° Celsius. These cells were filtered and cell culture was performed in Dulbecco's modified Eagle's medium containing 1% penicillin/streptomycin solution and 10% foetal bovine serum (all from FBS, Viralex, PAA Laboratories, Linz, Austria). Medium was changed every 2 to 3 days and cells were expanded through two passages by detachment using 0.25% trypsin, followed by resuspension, collection, and seeding in new culture flasks at a ratio of 1 : 3.

### 2.2. Immunofluorescence Staining for Desmin

**Figure 1:**
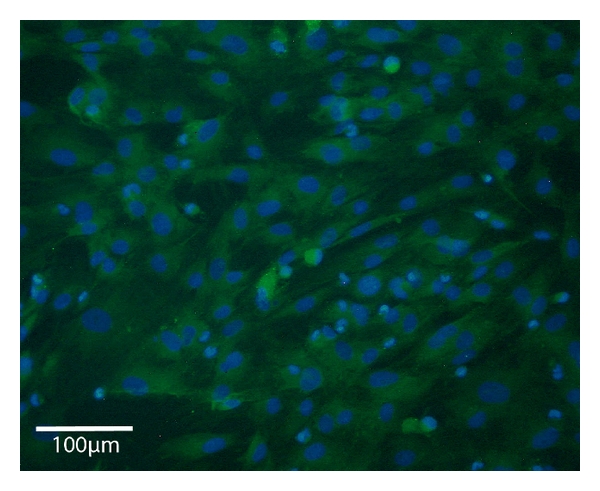
Immunofluorescence, 200*x*. 2D-culture of myoblasts harvested from 5 days old male Wistar rats: Desmin (green), nucleic counterstain with DAPI (blue).

The myogenic specificity of harvested myoblasts was verified by Desmin- and MyoD-immunocytochemistry (see Figure 1 (Desmin) before transplantation and Figure 6 (MyoD) after explantation). Therefore, the cell cultures were fixed in 100% methanol for 20 minutes at −20° Celsius, washed with (PBS Biochrom, Berlin, Germany), and permeabilized with TRITON X-100 (Sigma-Aldrich, Irvine, UK) 0.25% in PBS for 30 minutes. The cultures were then incubated with blocking solution (5% goat serum and 0.1% TRITON X-100 in PBS) for 30 minutes. After being washed with PBS, the cultures were incubated with rabbit anti-Desmin polyclonal antibody (Sigma-Aldrich, Irvine, UK). The cultures were then washed with PBS and incubated with anti-rabbit, IgG for 2 hours diluted 1 : 1500 in blocking solution. Finally nuclear counterstaining with DAPI (Sigma Aldrich, Irvine, UK) was performed through incubation with DAPI 10 *μ*g/1 mL aqua bidest for 30 minutes at room temperature followed by washing steps. After being mounted with Mowiol (Sigma-Aldrich, Irvine, UK), the Desmin-stained cultures were viewed and photographed by fluorescence microscopy (Carl Zeiss MicroImaging, Jena, Germany).

### 2.3. Lentiviral Production and Infection (Luciferase)

For the production of lentivirus, 7 × 10^6^ HEK 293T cells were seeded onto a 15 cm Petri dish and transfected after 24 h with jetPEI (PEQLAB) using 9.4 g of transfer vector pEMW-Luc (containing the coding sequence of Luciferase), 6.1 g of psPAX2 (packaging plasmid), and 3.3 g of pMD2. G (VSV-G envelope). Lentivirus were harvested 48 h after transfection, passed through a 0.22 *μ*m filter, and concentrated by ultracentrifugation at 26000 rpm for 2 h at 4°C. Viral particles were resuspended in PBS and stored at −80°C. For infection with the lentiviral stock, primary myoblasts were seeded in 10-cm plates in complete medium and infected with viral particles (MOI 5) and 8 g/L polybrene for 16 h at 37°C. Media were changed to growth media without polybrene. The average percentage of success of the transfected myoblasts was 89.4 percent in our efficacy tests.

### 2.4. Surgical Procedure

Male Wistar rats (Charles River, Kisslegg, Germany) at the age of 4 to 5 weeks served as recipients of the *in vivo* bioreactor chamber. German regulations for the care and use of laboratory animals have been observed at all times. All experimental protocols were approved by the governmental review committee of Northrhine-Westfalia, Germany. The animals were housed in the veterinary care facility of the RWTH University Hospital under temperature-controlled conditions at 21 ± 1°C with a cycle of 12-hour light and 12-hour darkness as well as free access to standard food and water.

**Figure 2:**
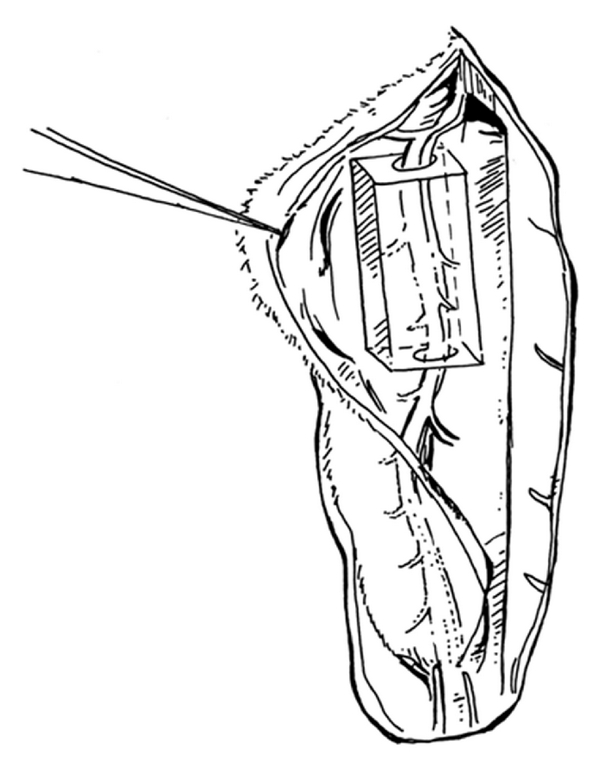
Implantation site showing the rectus abdominis dissected free with the superior and inferior epigastric vascular pedicle along the deep surface of the muscle. The *in vivo* bioreactor chamber is assembled around the epigastric vessels.

**Figure 3:**
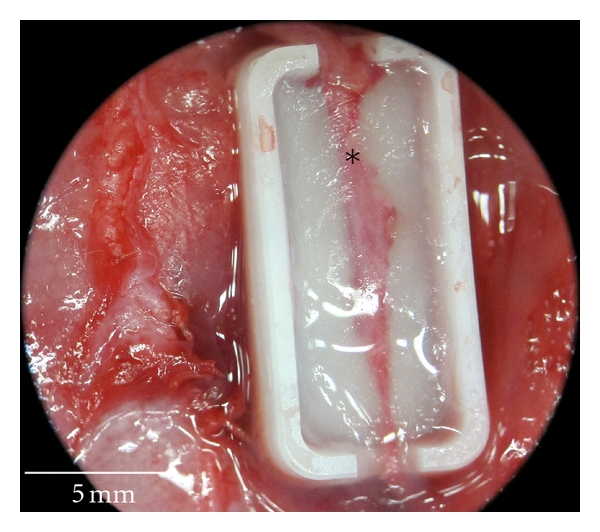
The implanted *in vivo* bioreactor chamber (outer width 10 mm, length 20 mm, height 6 mm) in a 4-weeks-old male Wistar rat with the epigastric artery (*) as central-core vessel; the transplanted and luciferase-transfected myoblasts are embedded in a fibrinogen matrix.

 Operations were performed by the same microsurgeon under an operative microscope (Karl-Zeiss, Jena, Germany). Anesthesia was induced with inhalative Isoflurane (Baxter, Unterschleißheim, Germany) and maintained by intraperitoneal injection of Ketamine (10%, 0.6 mL/kg) and Medetomidine (0.3 mL/kg; Pfizer, Paris, France). Ketoprofen (5 mg/kg) was administered preoperatively for analgesia. The surgical site was shaved, prepped, and draped for sterility. A mid-line incision was made and perforating branches to the cutaneous tissue were cauterized. The dissection of the rectus abdominis muscle was performed along the linea alba as described by Zhang et al. [18] starting retrograde from 2 cm above the symphysis pubis to the xyphoid (see Figure 2). Furthermore, for the group with perfused BAT (*n* = 4), the epigastric artery and vein were dissected carefully from the rectus abdominis muscle with a total length of at least 3 cm. The lower part of the polyoxymethylene (POM) customized bioreactor chamber (outer width 10 mm, length 20 mm, height 6 mm) was positioned under the epigastric vessels using these vessels as central-core vessels of the *in vivo* chamber. Fibrin glue was administered as matrix (Baxter, Unterschleißheim, Germany) embedding the epigastric vessels in the bioreactor, and 4 × 10^6^ myoblasts were suspended and mixed with the fibrin glue subsequently to complete the bioartificial tissue (see Figure 3). The bioreactor chamber was closed by attaching the upper part. The blood flow of the epigastric artery was checked by Doppler sonography proximal and distal of the chamber to ensure the patency before wound closing. Subsequently, the rectus abdominis muscle was adapted using Prolene 5-0 (Ethicon, Norderstedt, Germany) to achieve stability of the abdominal wall and protection of the bioreactor chamber. Finally, the wound was closed using Vicryl 5-0 (Ethicon, Norderstedt, Germany). In the control group with unperfused BAT (*n* = 3), the bioreactor chamber was implanted as described but without preparation and use of the epigastric vessels. For the explantation of the bioreactor chamber, the animals were reanaesthetized as described before on day seven, and after exposing the chamber, the epigastric artery was ligated and transected proximal and distal of the chamber allowing to remove the bioreactor completely without manipulation. After this explantation on day seven, the animals were sacrificed by intracardiac potassium chloride injections.

### 2.5. Bioluminescence Imaging

The followup of the transplanted myoblasts inside the *in vivo* bioreactor chamber was assessed by means of bioimaging with an ultrasensitive Xenogen camera (IVIS Lumina, Caliper Life Sciences, MA, USA) using the reporter D-Luciferin at different time points (d0, d1, d7). The animals were anesthetized with 2% Isoflurane and placed in the imaging chamber. After acquisition of a baseline image, the rats were injected with the substrate Luciferin intraperitoneally in order to induce photons (green light emission at 562 nm). The region of interest (ROI) was defined as the site where the *in vivo* bioreactor chamber is located. Peak signal (photons/second [p/s]) from that fixed ROI was evaluated using the Living Image 2.50 software (Xenogen). The background signal was evaluated at different locations not close to the bioreactor chamber as well as at different time points (d0, d1, d7) and subtracted from the corresponding peak signal.

### 2.6. Immunofluorescence Staining for MyoD (after Explantation)

Cryosections of explanted bioartificial tissue containing myoblasts of d7 were washed with PBS. The cultures were then incubated with blocking solution (5% goat serum and 0.1% TRITON X-100 in PBS) for 10 min. After being washed with PBS, the cultures were incubated with mouse anti-MyoD monoclonal antibody (Thermo Scientific, Astmoor, UK), diluted 1 : 50 in blocking solution (2 h at 20°C), and again washed with PBS and incubated with anti-mouse IgG (KPL, MD, USA) for 2 h diluted 1 : 100 in blocking solution. After being washed with PBS and mounted with Mowiol, anti-MyoD-stained cultures were viewed and photographed by fluorescence microscopy.

## 3. Results and Discussion

### 3.1. Myoblasts Can Be Luciferase-Transfected and Therefore Monitored *In Vivo* with Bioluminescence

**Figure 4:**
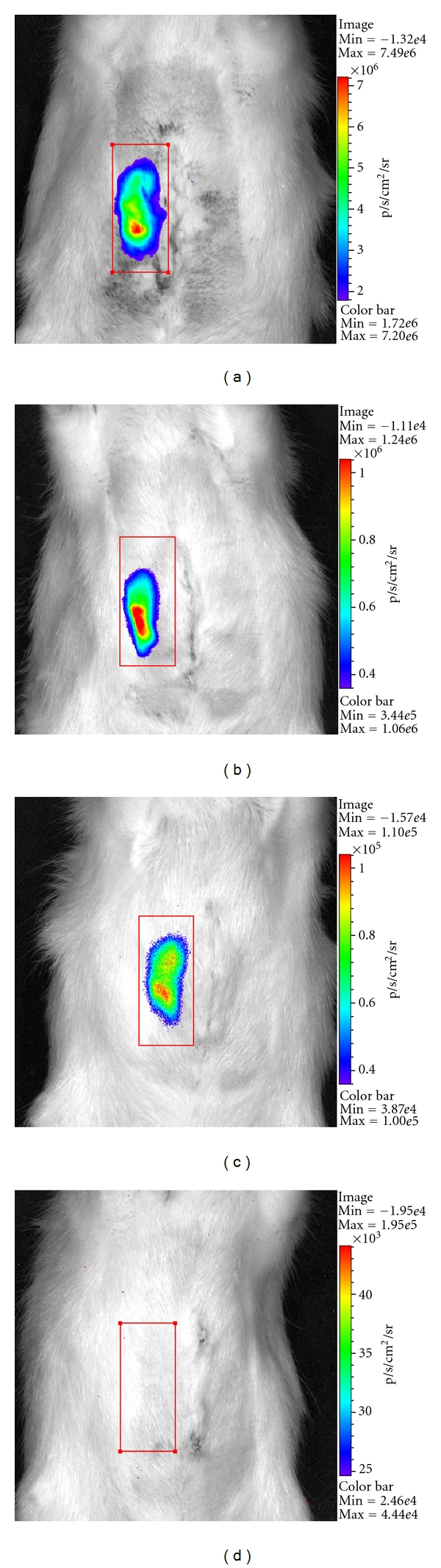
Bioluminescence followup after myoblasts implantation including the epigastric artery as central-core vessel (perfused BAT): (a) day of surgery, (b) 1 day after implantation, (c) 7 days after implantation, (d) 7 days after implantation of myoblasts without central-core vessel in 3D-construct (unperfused BAT).

**Figure 5:**
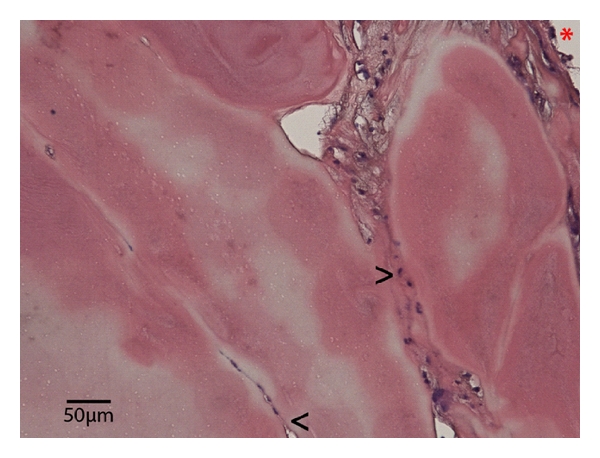
H&E staining of an explanted perfused BAT seven days after implantation showing the lumen of the epigastric artery (*), myoblasts inside the fibrin matrix (**>**) as well as starting capillarization within the matrix (**<**).

The bioluminescence followup monitoring of the Luciferase-transfected myoblasts showed the expected results with a high number of photons per second on day 0 directly after surgery (1.769 × 10^8^ photons/sec, see Figure 4 and Table 1) and exponentially decreasing bioluminescence signals on day 1 and 7 (3.426 × 10^7^ photons/sec and 3.031 × 10^6^ photons/sec) indicating the remission of the myoblasts cell survival. The evaluated background signals (4.822 × 10^5^ photons/sec) showed no significant differences in terms of location and time points. However, in unperfused BATs without arterial inflow, and therefore, no possible Luciferin supply, only the background signals (e.g., 4.110 × 10^5^ photons/sec on day 0) could be detected as well. Furthermore, the circumstance of retrieving bioluminescence signals in the perfused BATs (see Figures 4(a)–4(c)) and only background signals in the unperfused BATs (see Figure 4(d)) was approving the reliable patency of the epigastric artery as central-core vessel (see * at Figure 5) inside the BAT by providing the Luciferin-substrate via blood flow to the BAT. Therefore, this animal model offers both an *in vivo* bioreactor model for musculoskeletal or other different cell types including a central anastomosable core vessel as well as the possibility of monitoring the transplanted cells, in our study transfected myoblasts *in vivo*. Thus, the required animal number can be reduced, as there is no exceeding animal sacrifice necessary at different time points.

### 3.2. Capillarization within the Perfused BAT

**Figure 6:**
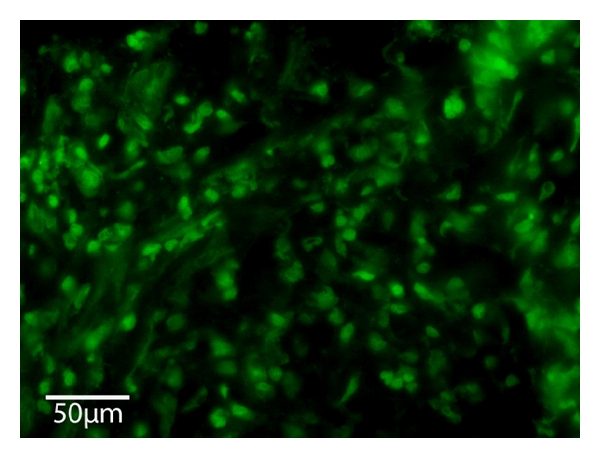
Immunofluorescence, 400*x*. Explanted perfused BAT seven days after transplantation showing positive MyoD staining for myoblasts.

Pulsatile perfusion within a 3-dimensional bioartificial tissue construct is promoting the capillarization [8, 9]. In our study, we could show that the epigastric artery is applicable as central-core vessel. Nevertheless, we could also show that seven days after transplantation there was a beginning capillarization within the BAT, in the areas close to the core vessel (see < at Figure 5). Further we could also prove the presence of myoblasts with positive MyoD staining by immunofluorescence (see Figure 6) after explantation on day seven showing that the myoblast phenotype persisted consistently. However, improvement of the matrix, for example, by inserting synthesized polymer fibers to achieve a more muscle-fiber-like structure will be needed to achieve both, a long-time stability as well as an organized histomorphological pattern of the myoblasts to get one more step closer to the use of bioartificial tissue in reconstructive surgery.

## 4. Conclusions

Myoblasts can be transfected with Luciferase and, therefore, bioluminescence is an effective and reliable method for assessment of myoblasts cell graft survival *in vivo*. The implantation of the *in vivo* bioreactor with the epigastric artery as core vessel was performed routinely with regular patency of the vessel and without any side effects or wound problems during the experimental period. Again, starting capillarization was found in the areas close to the core vessel. Thus, the epigastric artery is applicable as central-core vessel of the 3-dimensional construct and could later be also used as an anastomosable vessel of artificial musculoskeletal tissue for further experimental reconstructive surgeries after optimizing both matrix and cell survival. However, with the use of a predefined customized *in vivo* bioreactor chamber, this model offers also a variety of possible applications to create vascularized artificial tissue.

## Figures and Tables

**Table 1:** Bioluminescence imaging of the BATs perfused (*n* = 4) and unperfused (*n* = 3) on day 1, 3, and 7 after myoblasts-transplantation in photons/second and standard deviation (SD). As no substrate (Luciferin) can reach the transfected myoblasts within the unperfused BAT inside the bioreactor chamber, only background signals can be detected.

	Perfused BAT	Unperfused BAT
	photons/sec ± SD	

d0	1.769 × 10^8^ ± 1.576 × 10^7^	4.110 × 10^5^ ± 3.519 × 10^4^
d1	3.426 × 10^7^ ± 2.414 × 10^6^	5.172 × 10^5^ ± 5.822 × 10^4^
d7	3.031 × 10^6^ ± 4.087 × 10^5^	4.652 × 10^5^ ± 7.527 × 10^4^
